# Vitamin D Supplementation Reduces Intimal Hyperplasia and Restenosis following Coronary Intervention in Atherosclerotic Swine

**DOI:** 10.1371/journal.pone.0156857

**Published:** 2016-06-06

**Authors:** Gaurav K. Gupta, Tanupriya Agrawal, Vikrant Rai, Michael G. Del Core, William J. Hunter, Devendra K. Agrawal

**Affiliations:** Department of Clinical and Translational Science, Creighton University School of Medicine, Omaha, NE, United States of America; University of Central Florida, UNITED STATES

## Abstract

Vitamin D is a fat-soluble steroid hormone that activates vitamin D receptor to regulate multiple downstream signaling pathways and transcription of various target genes. There is an association between vitamin D deficiency and increased risk for cardiovascular disease. However, most of the studies are observational and associative in nature with limited data on clinical application. Thus, there is a need for more prospective randomized controlled studies to determine whether or not vitamin D supplementation provides cardiovascular protection. In this study, we examined the effects of the deficiency and supplementation of vitamin D on coronary restenosis following coronary intervention in atherosclerotic Yucatan microswine. Twelve Yucatan microswine were fed vitamin D-deficient (n = 4) or -sufficient (n = 8) high cholesterol diet for 6-months followed by coronary intervention. Post-intervention, swine in the vitamin D-sufficient high cholesterol diet group received daily oral supplementation of either 1,000 IU (n = 4) or 3,000 IU (n = 4) vitamin D3. Six months later, optical coherence tomography (OCT) was performed to monitor the development of intimal hyperplasia and restenosis. Animals were euthanized to isolate arteries for histomorphometric and immunohistochemical studies. Animals had graded levels of serum 25(OH)D; vitamin D-deficient (15.33 ± 1.45 ng/ml), vitamin D-sufficient + 1,000 IU oral vitamin D post-intervention (32.27 ± 1.20 ng/ml), and vitamin D-sufficient + 3,000 IU oral vitamin D post-intervention (51.00 ± 3.47 ng/ml). Findings from the OCT and histomorphometric studies showed a decrease in intimal hyperplasia and restenosis in vitamin D-supplemented compared to vitamin D-deficient swine. Vitamin D supplementation significantly decreased serum levels of TNF-α and IFN-γ, upregulated serum levels of IL-10, and had no effect on serum IL-6 levels. These findings suggest that vitamin D supplementation limits neointimal formation following coronary intervention in atherosclerotic swine and provide the support for vitamin D supplementation to protect against the development of coronary restenosis.

## Introduction

Coronary artery disease (CAD), the most common among cardiovascular diseases, is a primary cause of morbidity and mortality in the developed world and predicted to remain so for the next 20 years [1]. Although, percutaneous transluminal coronary angioplasty (PTCA) is safe and an effective means to treat CAD, development of post-angioplasty restenosis is a major limitation of this procedure, and occurs in 30–50% of patients [2–4]. Due to very high incidence of restenosis following balloon angioplasty, the majority of patients undergoing coronary intervention today are deployed with stents. The incidence of restenosis is reduced to 25% in the patients treated with balloon angioplasty with bare-metal stenting compared to balloon angioplasty alone [5, 6]. More recently, stents coated with anti-proliferative and anti-inflammatory agents, “drug-eluting” stents (DESs), have been used to prevent restenosis. Despite all of these benefits, the safety of DES has been called into question by recent studies, suggesting that DES could produce adverse arterial responses, including delayed endothelialization and hypersensitivity to the polymeric coating responsible for the regulation of drug dosing and release kinetics [7–10]. Several reports highlight the role of inflammation in the development of restenosis after percutaneous coronary interventions [11, 12]. Macrophages, vascular smooth muscle cells (VSMCs) and endothelial cells produce inflammatory cytokines [13–15] involved in the process of atherosclerosis. These cytokines include tumor necrosis factor (TNF)-α, interferon (IFN)-γ and interleukin (IL)-6, which induce the inflammatory response, cell proliferation and apoptosis, and thus leading to the pathogenesis of restenosis following PTCA.

Widespread prevalence of vitamin D deficiency and cardiovascular diseases, in conjunction with the higher incidence of ischemic heart disease, has been noted in countries with lower levels of ultraviolet-B light exposure [16]. There is seasonal variation in the levels of vitamin D with higher levels in the summer [17], and this correlates with the seasonal pattern of the incidence of ischemic heart disease [18]. Such evidence has encouraged to elucidate the possible association of vitamin D with cardiovascular disease. A significant association has been reported between the deficiency of serum 25(OH)D and cardiovascular disease risk factors, including obesity [19], metabolic syndrome [20], glucose intolerance [19], and hypertension [21]. Vitamin D supplementation has also been shown to reduce cardiovascular disease in the general population [22]. In another recent study it was concluded that the levels of vitamin D are linked with the incident of cardiovascular disease. The potential underlying mechanisms could include the inhibition of renin gene expression by 1,25(OH)2D3 and the potential role of vitamin D in vascular functions, such as smooth muscle cell proliferation, thrombosis and inflammation [23]. The possibility of the immunomodulatory effects of vitamin D was proposed based on the observation that vitamin D receptor (VDR) is significantly expressed in various immune cells, including, leukocytes, peripheral blood monocytes, antigen-presenting cells and activated CD4^+^ lymphocytes [24, 25]. The 1, 25(OH)2D3 interferes with nuclear factor-kappa B (NF-kB)- induced transcription of IL-12 and thus inhibits the production of IL-12 [26, 27]. On the contrary, 1, 25(OH)2 D3 upregulates the production of dendritic cell (DC)-derived IL-10, promoting Th2 cell phenotype [27, 28]. IL-10 may act in an autocrine manner to induce regulatory T-cells and inhibits production of pro-inflammatory cytokines, including TNF-α, IL-1, and IL-6. Furthermore, 1, 25(OH)2D3 also attenuates the function of differentiated CD4+ Th1 lymphocytes to synthesize IFN-γ [29].

Recent clinical studies support a relationship between serum vitamin D status and CAD severity [30–32]. However, the association of endogenous 25(OH)D levels with cardiovascular disease events following coronary intervention is still unknown and prospective studies are required to investigate whether vitamin D supplementation plays a role in cardiovascular protection post-coronary intervention. Proliferation of VSMCs and inflammation are the major pathological processes involved in the development of coronary restenosis. We have recently reported the decreased expression of VDR in the neointimal lesions after coronary intervention, suggesting a potential role of vitamin D in the pathogenesis of intimal hyperplasia [33]. There has been no prospective study investigating the effect of vitamin D status, deficiency, sufficiency and supplementation, on coronary restenosis. In this study, we designed experiments to examine the effect of vitamin D supplementation on the inflammation and the patency of coronary arteries following coronary intervention in a well-controlled atherosclerotic swine model of coronary restenosis.

## Material and Methods

### Animals and Diets

Prior to the initiation of the study, an approval of the research protocol (IACUC #0831) was obtained from the Institutional Animal Care and Use Committee of Creighton University. Yucatan microswine of 30–40 lbs were obtained from Sinclair Laboratories, Columbia, MO, USA. During the course of the study, all animals were housed in the Animal Resource Facility of Creighton University, Omaha, NE and NIH standards and USDA guidelines were followed for their care and experimental protocol. Animals were fed 1–1.5 lb/animal/day of experimental diet.

Vitamin D-deficient high cholesterol swine diet (Harlan, USA) contained the following major ingredients: 19% casein “vitamin free”, 23.5% sucrose, 23.9% corn starch, 13% maltodextrin, 4% soybean oil, 4% cholesterol, 20% chocolate mix, and 10% cellulose. Vitamin D-sufficient high cholesterol diet (Harlan, USA) was prepared with the following major ingredients: 37.2% corn (8.5% protein), 23.5% soybean meal (44% protein), 20% chocolate mix, 5% alfalfa, 4% cholesterol, 4% peanut oil, 1.5% sodium cholate, and 1% lard. The swine in vitamin D-deficient group were fed vitamin D-deficient high cholesterol diet before (6 months) and after (6 months) the coronary intervention. However, the swine in vitamin D-sufficient group were fed vitamin D-sufficient high cholesterol diet before (6 months) coronary intervention. But, after the coronary intervention, the swine in vitamin D-sufficient group were fed vitamin D-sufficient high cholesterol diet supplemented with 1,000 IU/d vitamin D to bring serum vitamin D levels within the physiological range. The swine in the vitamin D-supplemented group following coronary intervention were supplemented with 3,000 IU/d vitamin D to increase the serum 25(OH)D levels above the physiological range. Based on our previous experience and other studies, 1,000 IU and 3,000 IU/day of oral vitamin D were added to the vitamin D sufficient high cholesterol diet to achieve the normal physiological range and supplemental range of serum vitamin D, respectively [34–36].

### Experimental Protocol

The effect of vitamin D supplementation on coronary restenosis was examined by feeding the Yucatan microswine with vitamin D-deficient high cholesterol diet (4 animals) or vitamin D-sufficient high cholesterol diet (8 animals). After 6 months, the coronary intervention (balloon angioplasty and bare-metal stenting) was performed in all animals. Steps to prevent the thrombosis were taken by administering aspirin (325 mg/day) and ticlopidine (250 mg/day) to all animals three days prior to the procedure. Then, the animals were fasted overnight, and transported to the cardiovascular surgical suite, where swine were sedated by intramuscular injection of 0.1 ml/kg telazol/xylazine. A catheter for intravascular fluid (Ringer Lactate) was placed in the ear vein. Cefazolin (3–5 mg/kg, intramuscularly) was administered to all animals to limit the possibility of infection. After intubation of the animals, general anesthesia was given using 4% isoflurane. During the surgical procedure, vital signs, including temperature, heart rate, oxygen saturation, and capillary refill time, were periodically monitored. Following the cut down and exposure of the femoral artery, a 7F catheter was introduced. In order to maintain the proper blood flow prior to catheter introduction, 100U/kg heparin was administered intravenously. A 6F guide catheter was inserted to angiographically visualize the coronary vessels. Balloon angioplasty and stenting were done in the left anterior descending (LAD) and left circumflex (LCX) arteries respectively. Following the completion of the procedure, the femoral artery was sutured and the leg incision was closed. The animal was then transferred for post-operative care. The pain reliever, buprenorphine (0.1–0.3 mg/kg *i*.*m*.), was administered to all animals. Once the animal gained sternal recumbency, the intravenous line was removed. The vital signs were regularly checked until the animal was conscious and able to walk. One day after the procedure, the animal was transported back to the animal facility. Post-coronary intervention, animals in the vitamin D-sufficient high cholesterol group were further divided into 2 groups and received the daily oral supplementation of 1,000 IU (4 animals) and 3,000 IU (4 animals) of vitamin D3, respectively. Vitamin D- deficient high cholesterol diet group (4 animals) remained on the same diet without any vitamin D supplementation. Angiogram and optical coherence tomography (OCT) imaging were performed at 6 months post-coronary intervention to quantify in-segment minimal luminal diameter and intimal hyperplasia. High dose of barbiturates (Beuthanasia-D, 0.1 ml/lb, *i*.*v*.) was administered to euthanize the animal and coronary arteries were dissected for histomorphometric and immunohistochemical examination.

### Coronary Angiography, Angioplasty and Optical Coherence Tomography

Access to the femoral artery in the leg was gained by using an introducer needle and subsequent placement of a 6F sheath introducer to hold the artery open and regulate bleeding. Following this, a guiding catheter was introduced and pushed to the coronary arteries. To maintain blood flow, systemic heparin (100U/kg) was administered. If the procedure exceeded 90 min, an additional 50U/kg heparin was administered. Following this, a guidewire was inserted through the guiding catheter into the coronary artery. The left coronary artery was visualized and recorded angiographically using a 6F JR4 catheter. Non-ionic contrast media, 5–7 ml 60%, was injected into the coronary arteries for fluoroscopic evaluation. All angiographic images were stored in the C-arm (OEC 9900 Elite Vas 8, GE Healthcare). Coronary angiography and OCT were used to assess lumen loss. The percutaneous transluminal coronary angioplasty [PTCA] (Voyager Abbott) or PTCA with bare-metal stent (Vision) was gently pushed, until the deflated balloon catheter was inside the coronary arteries. The balloon was then inflated to 10–15 atm pressures, depending on the vessel to produce injury in the coronary artery endothelial cells. Bare metal sterile stents (VISION, Abbott) of clinical "coronary-type" (3.0 mm x 15 mm diameter) were used. The stents were mounted on 5F or 6F noncompliant angioplasty catheters (Cook Inc.). Angiograms were performed to estimate the TIMI grade angiographic blood flow. Then, the catheter was removed and the femoral artery sutured followed by closure of leg incision.

The OCT imaging is useful to clearly visualize the stent apposition and neointimal coverage of the stent struts. Complete imaging was performed and recorded using the C7-XR OCT intravascular imaging system (St. Jude Medical, St. Paul, MN). Minimal luminal diameter, reference diameter, and percent diameter stenosis were calculated.

### Blood Draw

Blood was drawn from ear vein at baseline, 6-months and 12-months for measuring complete lipid profile, complete metabolic profile, complete blood count (CBC), C-reactive protein (CRP), and serum 25(OH)D levels. The serum levels of IL-6, IL-10, TNF-α, and IFN-γ were quantified using ELISA kits.

### Histomorphometric Analysis

Hearts were surgically removed and the coronary arteries were carefully dissected followed by embedding in the paraffin. Thin sections were cut and serial sections were obtained for every 200 μm segment spanning the entire length of the vessel. The tissue sections were stained with hematoxylin & eosin (H&E), Verhoeff-Van Gieson (VVG) and Mason’s trichrome stain. The percent restenosis was quantified by morphometric analysis by calculating the area of restenosis and neointimal formation in the luminal surface, internal elastic lamina (IEL) and the external elastic lamina (EEL). The morphometric analyses were performed using the ImageJ software (http://rsb.info.nih.gov/ij/) and the percent area stenosis was quantified within the lumen (LA) and the IEL using the formula: % area stenosis = [1-(luminal area/IEL area)] x 100.

### Immunohistochemistry and Immunofluorescence

The paraffin-fixed thin sections of post-angioplasty porcine coronary arteries were used for the immunohistochemistry and immunofluorescence studies by the methods established in our laboratory [37]. Deparaffinization and rehydration were done before staining the slides. Briefly the tissue sections were incubated overnight at 4°C with primary antibodies against smooth muscle actin-alpha (α-SMA) (Sc-58669) and anti-proliferative cell nuclear antigen (PCNA) (sc-25280; purchased from Santa Cruz Biotechnology). Next, tissue sections were incubated for 1 hour with biotinylated secondary antibody (VECTASTAIN Elite ABC system) or affinity purified goat anti-mouse cyanine 3 (cy3) secondary antibodies (Jackson ImmunoResearch, Westgrove, PA). The tissue sections incubated with the isotype primary antibody were processed in parallel to serve as negative controls. The immunopositivity to the proteins of interest in the tissue sections was visualized under a microscope and photographed with an Olympus DP71 camera.

### Enzyme-Linked Immunosorbent Assay (ELISA)

The ELISA kits for porcine IFN-γ, porcine IL-6, and porcine TNF-α (Ray Biotech, Inc., Norcross, GA) and swine IL-10 (eBioscience, San Diego, CA) were used to measure serum concentrations of IFN-γ, IL-6, TNF-α, and IL-10, according to the protocol of the manufacturer. Briefly, the 96-well plates were pre-coated with anti-porcine IFN-γ, anti-porcine IL-6, anti-porcine TNF-α, and anti-porcine IL-10 by the manufacturer. After adding 100μl serum in each well, the 96-well plates were incubated at room temperature for 2½ hrs, followed by four times washing with wash buffer. Then, the biotinylated monoclonal antibodies to porcine IFN-γ, IL-6, TNF-α, and IL-10 were added and the plates were kept for incubation with the antibodies for an hour at room temperature followed by addition of horseradish peroxidase-conjugated streptavidin and incubation for additional 45 min at room temperature. To quantify the levels of the cytokines in each serum sample, a standard curve for the recombinant porcine IFN-γ, IL-6, TNF-α, and IL-10 was constructed using the computer-generated four-parameter curve-fit for each cytokine to determine the cytokine level in each sample.

### Statistical analysis

The values for the outcome measures are shown as mean± SEM. In order to determine the statistical significance between the experimental groups, statistical analysis was performed using GraphPad Prism 5.0 (GraphPad Software, Inc., San Diego, CA). Statistical significance between the experimental groups was determined by using two-tailed unpaired student *t*-test or one-way ANOVA with Bonferroni’s multiple comparison tests. The p<0.05 was considered as significant.

## Results

### Effect of Experimental Diets on Circulating Vitamin D Levels and Biochemical Parameters

**Fig 1:**
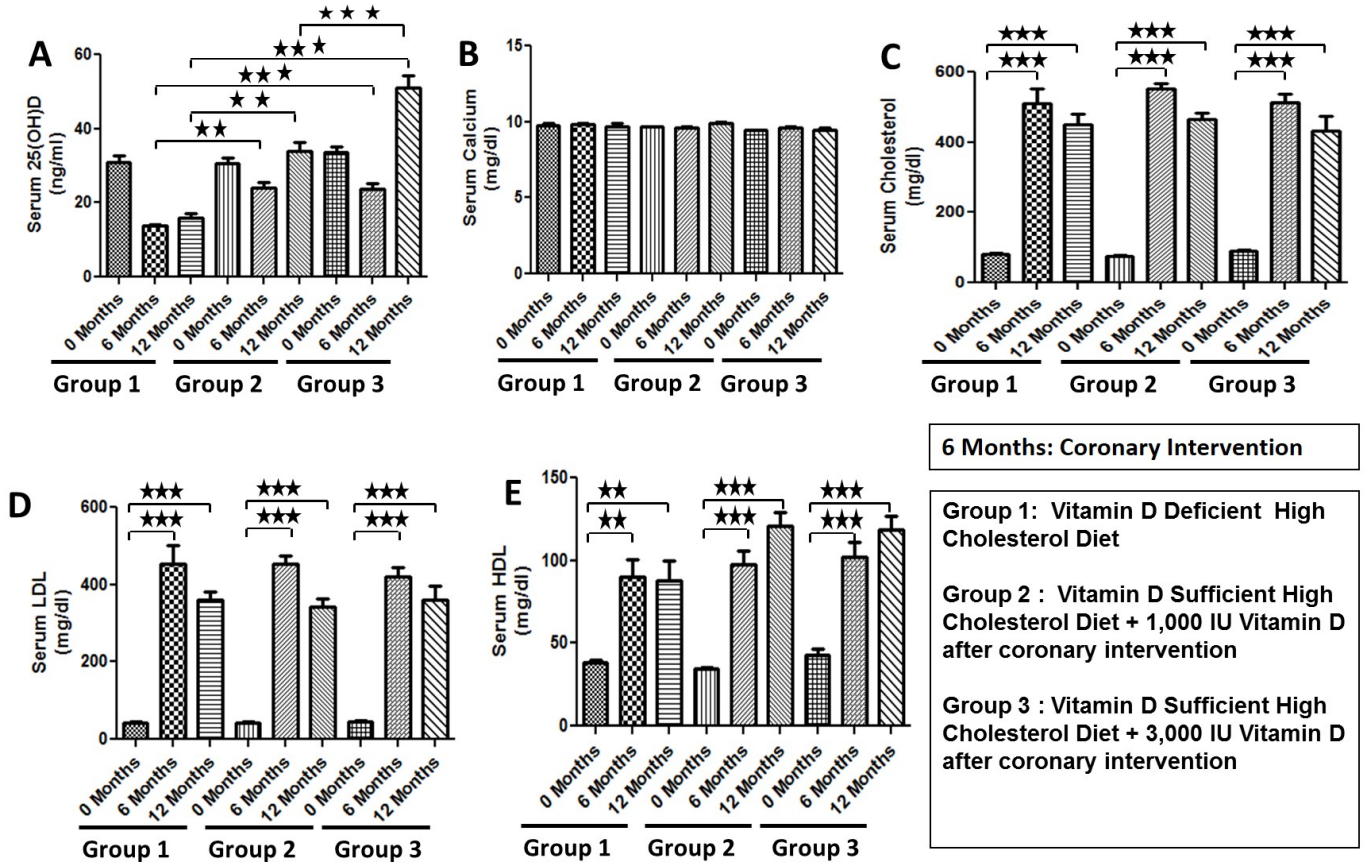
Effects of experimental diets on biochemical parameters. Data are shown on serum 25-hydroxy vitamin D (Fig 1A), serum calcium (Fig 1B), total serum cholesterol (Fig 1C), low density lipoprotein (LDL) (Fig 1D), and high density lipoprotein (HDL) (Fig 1E) levels of the female Yucatan microswine following a total of 12 months administration of the diet. Group 1: Vitamin D-deficient high cholesterol diet; Group 2: Vitamin D-sufficient high cholesterol diet + 1,000 IU/d vitamin D after coronary intervention; Group 3: Vitamin D-sufficient high cholesterol diet + 3,000 IU/d vitamin D after coronary intervention; Data are shown as mean ± SEM (N = 4 in each experimental group); **p< 0.01, ***p< 0.001.

Vitamin D-deficient high cholesterol diet induced vitamin D deficiency in swine. A serum level of 25(OH)D, the major circulating form of vitamin D, was significantly decreased in swine on vitamin D-deficient high cholesterol diet (15.33 ± 1.45 ng/ml) compared to swine on vitamin D-sufficient high cholesterol diet+ 1,000 IU oral vitamin D post-intervention (32.27 ± 1.20 ng/ml), and vitamin D-sufficient high cholesterol diet+ 3,000 IU oral vitamin D post-intervention (51.00 ± 3.47 ng/ml) (Fig 1A). Since all swine were kept in the controlled environment, there was an initial decrease in the serum levels of 25(OH)D due to controlled diet and dark conditions. However, after 6 months, the swine fed the vitamin D sufficient high cholesterol diet with vitamin D supplementation of 1,000 IU/d and 3,000 IU/d showed increase in the serum levels of 25(OH)D. There was no effect of vitamin D deficiency on serum calcium levels (Fig 1B). As expected, high cholesterol diet induced the severe hypercholesterolemia in all animals with no significant differences in total serum cholesterol, high-density lipoprotein (HDL) and low-density lipoprotein (LDL) levels between the groups (Fig 1C–1E).

### Effect of Vitamin D Supplementation on the Degree of Restenosis Following Coronary Intervention

**Fig 2:**
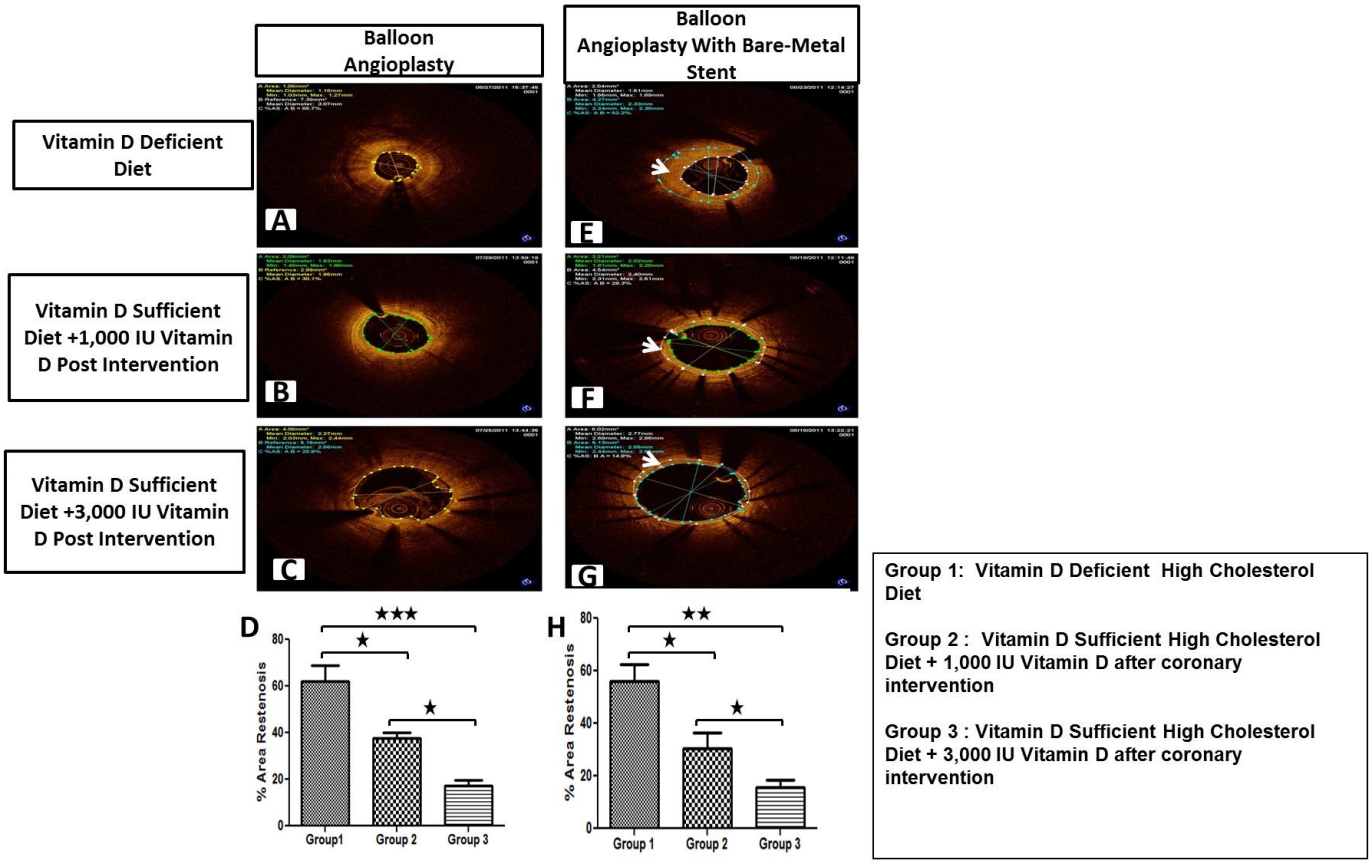
OCT examination of six months post intervention porcine coronary arteries—Representative images. Coronary arteries at the balloon angioplasty site (A-C) (E-G) and coronary arteries following bare metal stenting (E-G). Quantitative analysis was performed to calculate % area restenosis in post-angioplasty (D) and post-stenting (H) coronary arteries (N = 12). Group 1: Vitamin D-deficient high cholesterol diet; Group 2: Vitamin D-sufficient high cholesterol diet + 1,000 IU/d vitamin D after coronary intervention; Group 3: Vitamin D-sufficient high cholesterol diet + 3,000 IU/d vitamin D after coronary intervention; Data are shown as mean ± SEM (N = 4 in each experimental group); *p< 0.05, **p< 0.01, ***p< 0.001.

Six months following the experimental diets, animals underwent either balloon angioplasty alone or balloon angioplasty with bare-metal stenting. Coronary angiograms were obtained at baseline, and at 6-months follow-up. Optical coherence tomography (OCT) and histomorphometric analyses were performed to evaluate the development of neointimal hyperplasia and restenosis. As shown in Fig 2, the OCT images obtained post-coronary intervention at 6-months follow-up revealed the development of post-angioplasty restenosis (Fig 2A–2C) and in-stent restenosis (Fig 2E–2G). The degree of post-angioplasty percentage area restenosis was greater in vitamin D-deficient high-cholesterol group (62.26 ± 6.67%) compared to vitamin D-sufficient high-cholesterol diet + 1,000 IU vitamin D supplemented (37.66 ± 2.06%) (p < .05) and vitamin D-sufficient-high cholesterol diet + 3,000 IU vitamin D supplemented (17.2 ± 2.59%) group (p < .001) (Fig 2D). The degree of in-stent restenosis showed the same pattern where vitamin D-deficient high-cholesterol diet group had higher percentage area restenosis (56.23 ± 4.47%) compared to vitamin D-sufficient high-cholesterol + 1,000 IU vitamin D supplemented diet (30.66 ± 5.70%) (p < .05) and vitamin D-sufficient-high cholesterol + 3,000 IU vitamin D supplemented diet (15.53 ± 3.30%) groups (p < .01) (Fig 2H).

**Fig 3:**
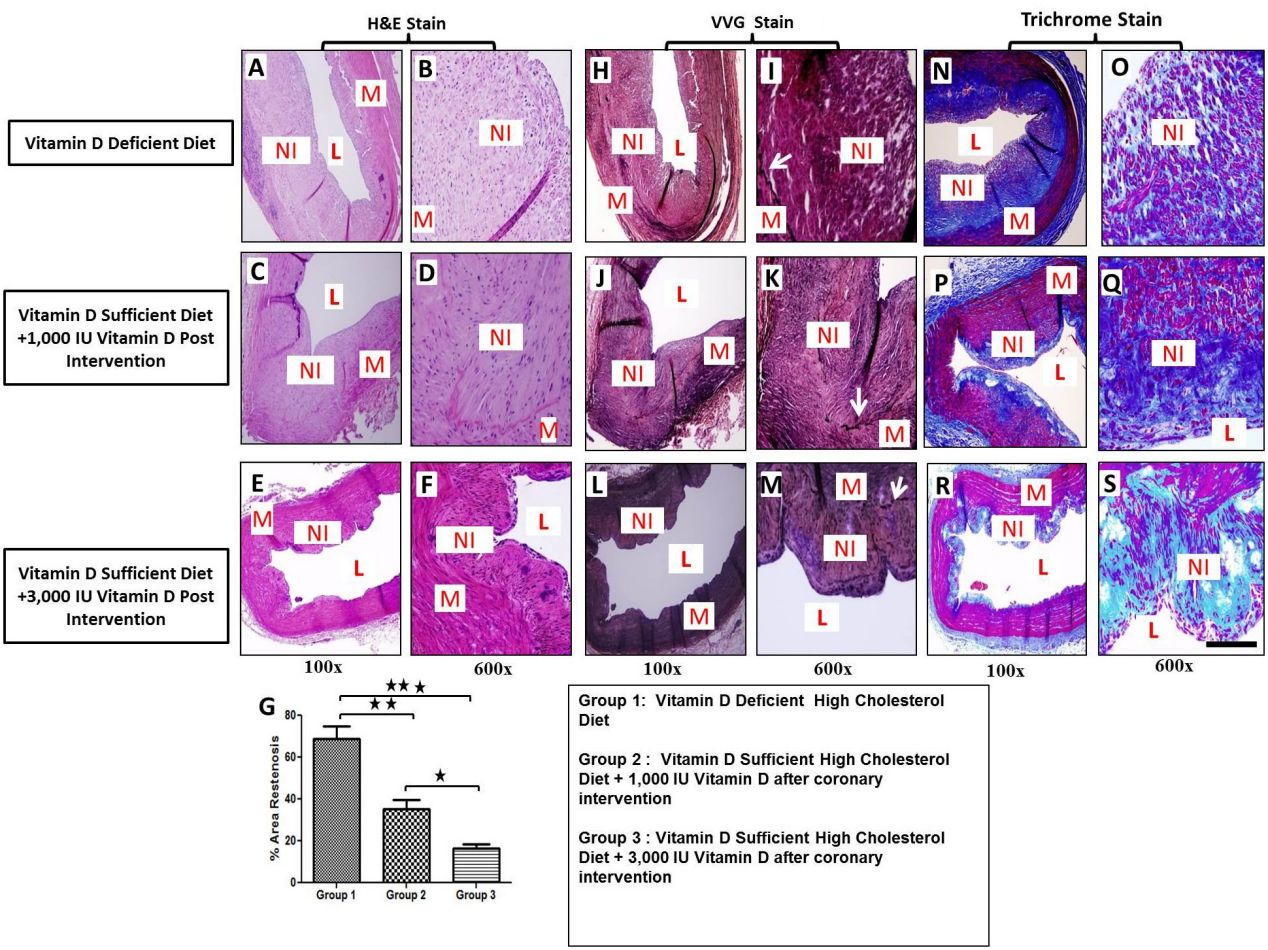
Effect of vitamin D supplementation on the development of restenosis following balloon angioplasty. Paraffin embedded thin sections were cut, deparaffinized and stained with hematoxylin and eosin (H&E), Verhoeff–Van Gieson stain (VVG), and Masson’s trichrome stain. H&E staining shows that the magnitude of the neointimal formation is higher in vitamin D-deficient high cholesterol diet group (A-B) compared to vitamin D-sufficient high cholesterol diet + 1,000 IU vitamin D group (C-D) and vitamin D-sufficient high cholesterol diet + 3,000 IU vitamin D supplemental group (E-F). VVG staining shows the disrupted IEL and neointimal thickening in all tissue sections (H-M). Masson’s trichrome staining revealed increased extracellular matrix material in the neointimal tissue of vitamin D-deficient high cholesterol diet group (N-O) compared to vitamin D-sufficient high cholesterol diet + 1,000 IU vitamin D group (P-Q) and vitamin D-sufficient high cholesterol diet + 3,000 IU vitamin D supplemental group (R-S). Quantitative analysis of % area stenosis (G); Magnification (100x–600x); Scale bar 100 μm; IEL: internal elastic lamina, L: lumen, M: medial layer, NI: neointima; Data are shown as mean ± SEM (N = 4); *p< 0.05, **p< 0.01, ***p< 0.001.

**Fig 4:**
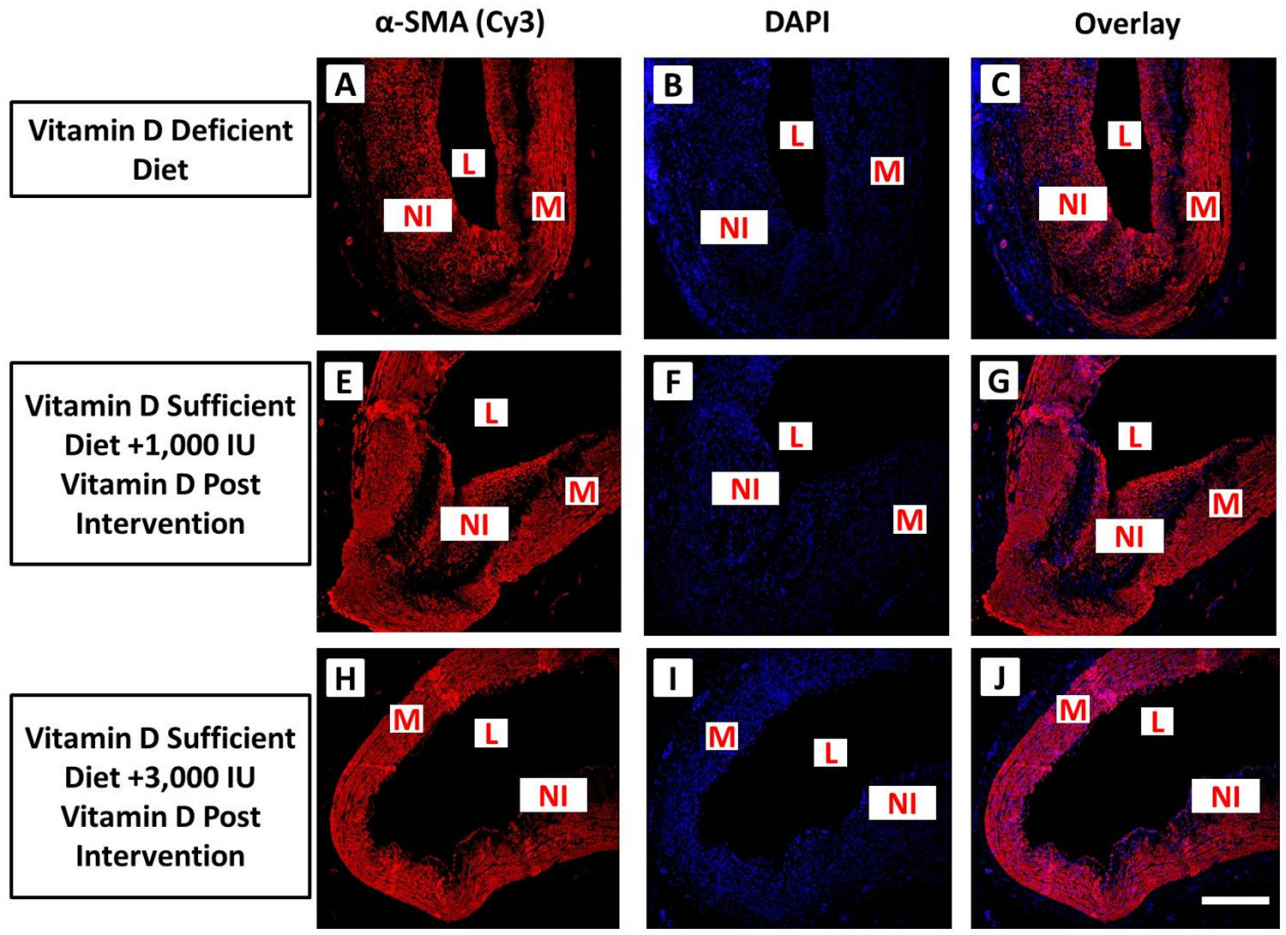
Immunofluorescence of α-SMA expression in post balloon angioplasty coronary arteries in experimental groups. Vitamin D-deficient high cholesterol diet (panels A-C), vitamin D-sufficient high cholesterol diet + 1000 IU vitamin D group (panels E-G) and () vitamin D-sufficient high cholesterol diet + 3000 IU vitamin D supplemental group (panels H-J). Sections were stained using mouse anti-alpha smooth muscle actin (α-SMA) antibody and goat anti-mouse cy3 as secondary antibody (panels A, E, D). DAPI was used to stain the nuclei (panels B, F, I. DAPI overlay with mouse anti-α-SMA antibody and goat anti-mouse cy3 as secondary antibody (panels C, G, J). Strong expression of α-SMA was found in the neointimal area as well as in media. Scale bar 100 μm; L: lumen, M: medial layer, NI: neointima (N = 4).

The post-angioplasty vessels were examined by histomorphometric evaluation using H&E, VVG-elastin, and Masson’s trichrome staining. As shown in the representative photomicrographs of H&E stain (Fig 3A–3F), the percentage area restenosis was significantly greater in vitamin D-deficient high-cholesterol diet group (68.9 ± 6.06%) compared to vitamin D-sufficient high-cholesterol + 1,000 IU vitamin D supplemented diet post-angioplasty (35.4 ± 4.23%) (p <0.01) and vitamin D-sufficient-high cholesterol + 3,000 IU vitamin D supplemented diet post-angioplasty (16.4 ± 2.12%) groups (p< 0.001) (Fig 3G). VVG staining revealed injury due to balloon angioplasty and demonstrated rupture in the medial layer with clear disruption of the internal elastic lamina (IEL) in all coronary arteries post-angioplasty (Fig 4H–4M). The presence of abundant collagen in the neointima as well as in the adjoining adventitia was clearly evident in the tissue sections stained with Masson’s trichrome (Fig 3N–3S).

### Effect of Vitamin D Supplementation on Neointimal Formation and Cell Proliferation

**Fig 5:**
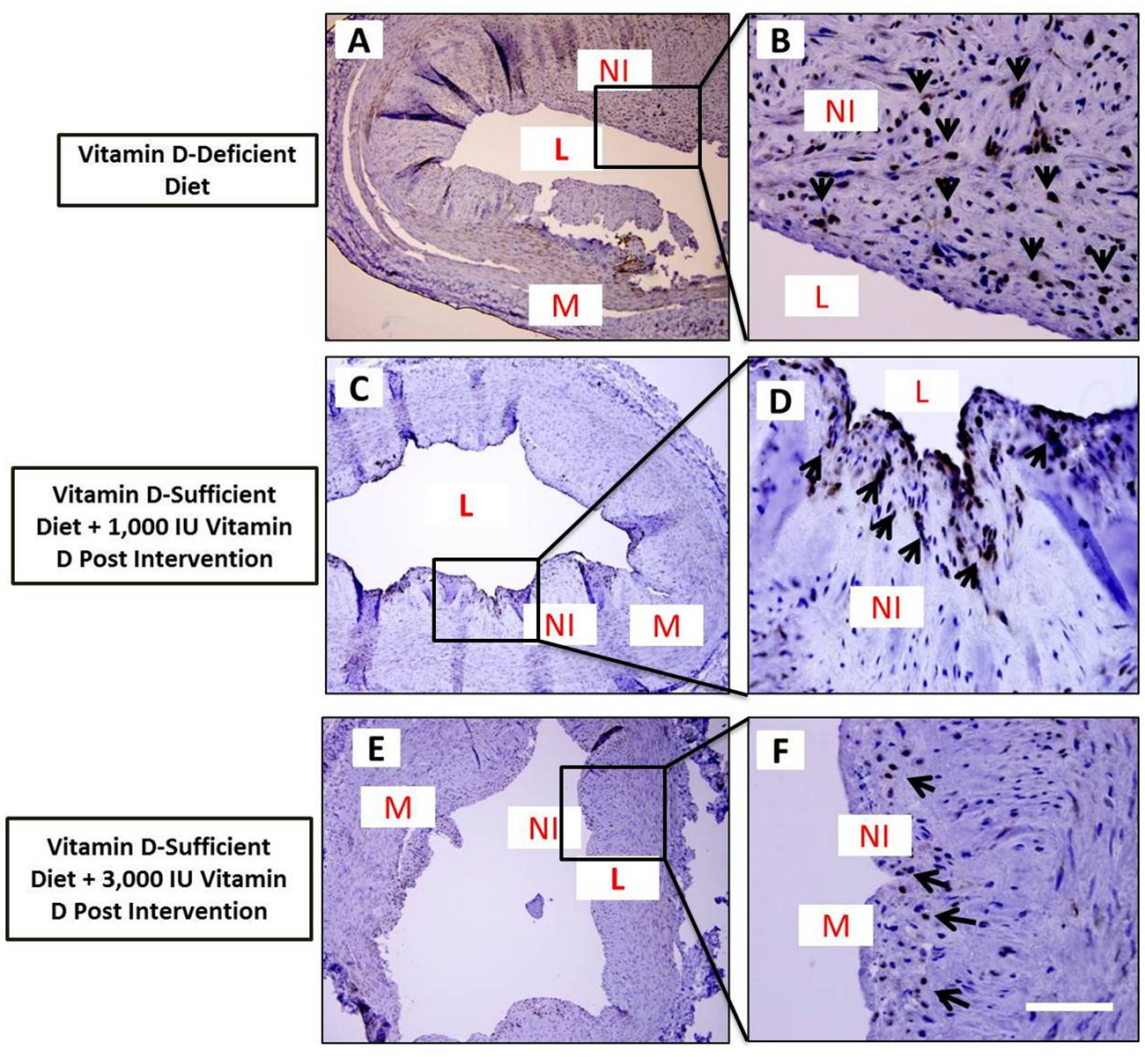
Effect of vitamin D supplementation on VSMC proliferation after balloon angioplasty. Photomicrographs showing immunohistochemical expression of proliferating cell nuclear antigen (PCNA) in thin sections of post-balloon angioplasty coronary arteries from vitamin D-deficient high cholesterol diet (panels A-B) and vitamin D-sufficient high cholesterol diet + oral vitamin D supplemental swine (panels C-F). Sections were stained with DAB as chromogen and counterstained using hematoxylin. Arrows indicate cells expressing PCNA. Scale bar 100 μm; L: lumen, M: medial layer, NI: neointima; Data are shown as mean ± SEM (N = 4); *p< 0.05, **p< 0.01, ***p< 0.001.

The effect of vitamin D supplementation on VSMC proliferation in post-angioplasty coronary arteries was examined by immunofluorescence of coronary artery tissue sections for α-SMA (Fig 4), a marker for VSMCs, and immunohistochemical staining of coronary artery tissue sections for PCNA (Fig 5), a marker of cell proliferation. Neointimal tissue predominantly comprised of α-SMA-expressing cells indicating that VSMCs are the major component of neointimal thickening developed following balloon angioplasty (Fig 4).

Significant number of cells expressed PCNA in neointimal tissue of the coronary arteries following angioplasty in the vitamin D-deficient high cholesterol diet group (Fig 5A and 5B). PCNA staining was primarily evident adjacent to the internal elastic lamina, suggesting that this area was the primary site of cell proliferation. In contrast, the immunopositivity of the PCNA staining was less in the neointima of vitamin D-sufficient high cholesterol diet + vitamin D supplemental diet groups (Fig 5C–5F). These data support the key effect of vitamin D on VSMCs proliferation, which might correlate with the observed decrease in neointimal formation.

### Effect of Vitamin D Supplementation on Serum Cytokines

**Fig 6:**
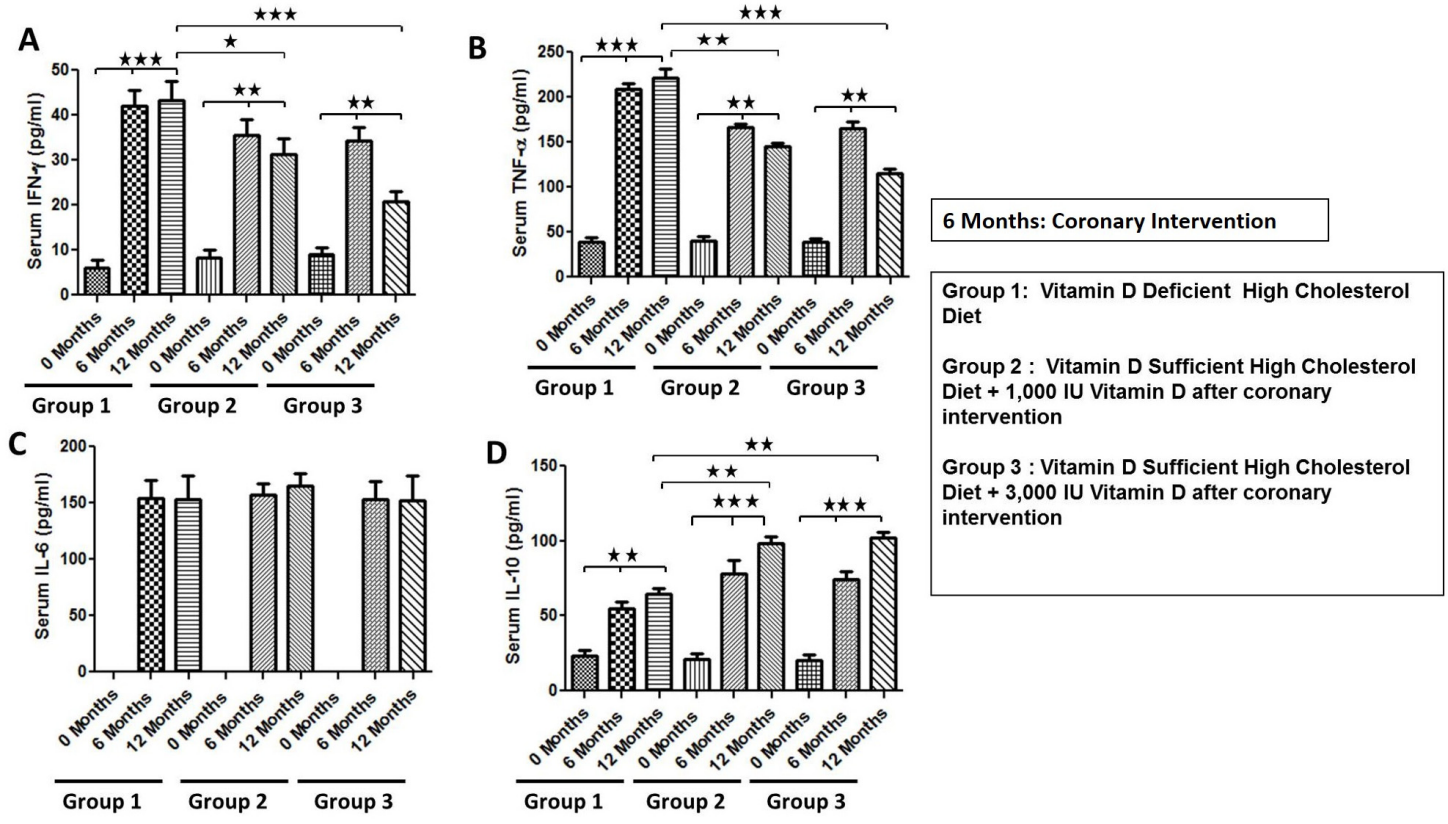
Effect of vitamin D on serum cytokine expression. Serum levels of IFN-γ (A), TNF-α (B), IL-6 (C), and IL-10 (D) were quantified using ELISA. Group 1: Vitamin D-deficient high cholesterol diet; Group 2: Vitamin D-sufficient high cholesterol diet + 1000 IU vitamin D after coronary intervention; Group 3: Vitamin D-sufficient high cholesterol diet + 3000 IU vitamin D after coronary intervention. Data presented as mean± SEM (N = 4); **p< 0.01, ***p< 0.001.

Next, the effect of vitamin D supplementation was investigated on the circulating levels of IFN-γ, TNF-α, IL-6, and IL-10 in the serum of atherosclerotic pigs with coronary intervention. The two doses of oral vitamin D (1,000 IU and 3,000 IU) were given in the post-coronary intervention phase. The serum specimens were collected for baseline pre- and 6-month post-coronary intervention and the cytokine levels were quantified using ELISA. The absolute mean values of all cytokines in the three experimental groups are shown in Fig 6. The basal level of serum IL-6 was below the detectable limits in all pigs. Hypercholesterolemic diet significantly upregulated the serum levels of all cytokines. In group 1 after 6 months of high cholesterol diet there was about 600%, 450%, 158%, and 152% increase, and after 12 months there was about 600%, 471%, 156% and 63% increase from the baseline in the levels of IFN-γ, TNF-α, IL- 6 and IL-10, respectively. In group 2 after 6 months of high cholesterol diet there was about 400%, 283%, 160%, and 285% increase, and after 12 months there was about 300%, 227%, 160% and 285% increase from the baseline in the levels of IFN-γ, TNF-α, IL- 6 and IL-10, respectively. In group 3 after 6 months of high cholesterol diet there was about 273%, 293%, 155%, and 270% increase, and after 12 months there was about 123%, 180%, 155% and 398% increase from the baseline in the levels of IFN-γ, TNF-α, IL- 6 and IL-10, respectively (Fig 6).

These data suggest that the vitamin D supplementation significantly downregulated the level of IFN-γ, TNF-α, upregulated IL-10 levels, and had no effect on serum IL-6 levels in the vitamin D-sufficient and vitamin D-supplemented swine (Fig 6).

## Discussion

There is a strong link between vitamin D deficiency and increased risk of the cardiovascular disease-related mortalities including congestive heart failure, hypertension, myocardial infarction, and peripheral arterial disease [38]. Most of the evidence to support this, however, have been derived from studies that are epidemiological in nature. Recent studies support the idea that several other indices of vascular function—including the development and progression of atherosclerosis—may be associated with vitamin D deficiency [39]. The precise mechanism by which vitamin D influences the development, progression and prognosis of CAD has yet to be explained. In addition, the clinical stage of CAD in which vitamin D could be most beneficial is unclear.

In this study, we for the first time investigated the *in vivo* effects of vitamin D status on the degree of neointimal hyperplasia following balloon angioplasty and intravascular stenting in coronary arteries. In our investigation on the protective effect of vitamin D against the neointimal response after vascular injury, we used a well-established swine coronary artery disease model. Pigs are widely recognized experimental model for the development of atherosclerosis and coronary artery diseases similar to human. The development of atherosclerosis in pigs can be spontaneously induced by feeding high fat diet [40]. The swine model of coronary restenosis closely resembles the proliferative component of restenosis in humans [41, 42]. In this model, we found significantly increased serum levels of lipid contents and atherosclerotic lesions were developed in coronary arteries of all pigs fed high cholesterol diet. Coronary intervention using balloon angioplasty provoked proliferation of VSMCs that resulted in neointimal hyperplasia in all coronary arteries. The neointimal tissue was primarily comprised of VSMCs, as demonstrated by positive staining for α-SMA.

Vitamin D exerts growth inhibitory and immunomodulatory effects that may potentially be useful in managing disorders such as atherosclerosis, post-interventional restenosis, and post-transplant vasculopathy, in which the underlying hallmarks of the pathological processes are remodeling in the vascular wall and uncontrolled cell growth. Indeed, 1,25(OH)_2_D_3_, the active form of vitamin D, and other agonist for VDR inhibit the proliferation of VSMCs [9]. Another study reported a dose-dependent inhibitory effect of 1,25(OH)_2_D_3_ on SMC proliferation in human coronary arteries [25]. The findings in this study support the hypothesis that there is an inverse correlation between the vitamin D status and the development and degree of neointimal hyperplasia in coronary arteries following balloon angioplasty. Additionally, the *in-vivo* anti-proliferative effect of vitamin D is supported by the finding of significantly reduced density of PCNA-positive cells in the neointimal region. This could be further substantiated by the effect of oral vitamin D supplementation after vascular injury. We found that the vitamin D supplementation significantly increased serum vitamin D levels in a dose-dependent manner. Increased levels of serum vitamin D are accompanied with the attenuation of neointimal tissue formation following balloon angioplasty with and without bare-metal stenting.

Inflammatory response and the proliferation of VSMCs have been implicated as critical underlying events in the development of restenosis. After coronary intervention, there is subsequent inflammatory response in injured vessels associated with increased production of inflammatory cytokines. In this study, we found increased levels of TNF-α, IL-6, INF-γ and IL-10 in association with neointimal formation and rupture of internal elastic lamina. The pro-inflammatory and apoptotic activity of TNF-α and anti-apoptotic activity of IL-10 have been previously described [43]. Furthermore, the role of TNF-α, IFN-γ, IL-1β, IL-6, IL-8, IL-4 and IL-10 in the pathogenesis of atherosclerosis in carotid artery post-carotid endarterectomy has been documented [44]. Higher circulating levels of TNF-α and lower circulating levels of IL-10 have been reported to be associated with higher probability of plaque formation and plaque burden in coronary arteries with plaque vulnerability [45]. Recently, another study has reported the potential role of TNF-α in pathogenesis of atherosclerosis and plaque formation and its role in plaque vulnerability in carotid arteries [46]. These studies suggest the critical role of pro-inflammatory cytokines in the plaque formation and blockade of the artery, and protective role of IL-10 in the pathogenesis of plaque formation. Increased levels of inflammatory cytokines with neointimal formation in our study support the role of inflammatory cytokines in restenosis after coronary intervention. In this study, it is noteworthy that vitamin D supplementation decreased the pro-inflammatory cytokines (TNF-α, IFN-γ and IL-6) and increased the levels of IL-10 from 6-months to 12-months period, suggesting a positive association between vitamin D supplementation and augmentation in the levels of IL-10. In addition to anti-proliferative effects, vitamin D may also reduce inflammatory milieu by suppressing pro-inflammatory cytokines, and by increasing the anti-inflammatory cytokines. In a randomized control trial in congestive heart failure patients, vitamin D supplementation significantly reduced the concentration of pro-inflammatory cytokine, TNF-α, with an upregulation in IL-10 levels [47]. The findings in another case-cohort study revealed a negative correlation between vitamin D status and the circulating levels of inflammatory cytokines in type 2 diabetic patients [48]. In accordance to previous studies, the results from our study demonstrated that vitamin D has immunomodulatory effects and obliterates the inflammatory cytokine expression. Supplementation of vitamin D inhibits the circulating levels of pro-inflammatory cytokines, including TNF-α and IFN-γ and increases the serum levels of IL-10, which may further contribute to the effective reduction of intimal hyperplasia and restenosis following vascular injury due to coronary intervention.

## Study Limitations

In this study, we primarily intended to investigate whether serum vitamin D levels affect the development and degree of intimal hyperplasia and restenosis following balloon angioplasty and stenting in atherosclerotic swine. However, the detailed underlying mechanisms of the beneficial effects of vitamin D warrant further studies.

## Conclusions

In summary, we found a significant reduction in the degree of intimal hyperplasia and restenosis in vitamin D-supplemented swine compared to the animals with deficient vitamin D status. Additionally, vitamin D supplementation significantly downregulated the serum level of pro-atherogenic cytokines, TNF-α and IFN-γ, and upregulated the serum levels of anti-inflammatory cytokine, IL-10. These novel findings suggest that vitamin D supplementation restricts the pathogenesis of neointimal formation in atherosclerotic swine and provides the support for using vitamin D, perhaps as an adjunct therapy, in protection against the development of intimal hyperplasia and coronary restenosis following coronary intervention.

## Disclaimer

The content of this article is solely the responsibility of the authors and does not necessarily represent the official views of the National Institutes of Health.
